# p120ctn and P-Cadherin but Not E-Cadherin Regulate Cell Motility and Invasion of DU145 Prostate Cancer Cells

**DOI:** 10.1371/journal.pone.0011801

**Published:** 2010-07-27

**Authors:** Sandra Kümper, Anne J. Ridley

**Affiliations:** Randall Division of Cell and Molecular Biophysics, King's College London, London, United Kingdom; University of Birmingham, United Kingdom

## Abstract

**Background:**

Adherens junctions consist of transmembrane cadherins, which interact intracellularly with p120ctn, ß-catenin and α-catenin. p120ctn is known to regulate cell-cell adhesion by increasing cadherin stability, but the effects of other adherens junction components on cell-cell adhesion have not been compared with that of p120ctn.

**Methodology/Principal Findings:**

We show that depletion of p120ctn by small interfering RNA (siRNA) in DU145 prostate cancer and MCF10A breast epithelial cells reduces the expression levels of the adherens junction proteins, E-cadherin, P-cadherin, ß-catenin and α-catenin, and induces loss of cell-cell adhesion. p120ctn-depleted cells also have increased migration speed and invasion, which correlates with increased Rap1 but not Rac1 or RhoA activity. Downregulation of P-cadherin, β-catenin and α-catenin but not E-cadherin induces a loss of cell-cell adhesion, increased migration and enhanced invasion similar to p120ctn depletion. However, only p120ctn depletion leads to a decrease in the levels of other adherens junction proteins.

**Conclusions/Significance:**

Our data indicate that P-cadherin but not E-cadherin is important for maintaining adherens junctions in DU145 and MCF10A cells, and that depletion of any of the cadherin-associated proteins, p120ctn, ß-catenin or α-catenin, is sufficient to disrupt adherens junctions in DU145 cells and increase migration and cancer cell invasion.

## Introduction

During metastasis, cancer cells commonly gain the ability to migrate and invade tissues by regulating their interaction with other cells and their extracellular environment. Many studies show that alterations in cell-cell adhesion correlate with epithelial tumour progression and metastasis [1]. Cancer cells can invade either as single cells or collectively as groups of cells [2],[3],[4]. Both types of invasion involve disruption of the epithelium which usually requires a weakening of cell-cell contacts and a change in cell shape. Cadherins and catenins form adherens junctions, which are central mediators of cell-cell adhesion. Expression of adherens junction proteins is often decreased in tumours, and reconstitution of functional adherens junctions can revert the invasive phenotype of cancer cells [5],[6],[7].

The classical cadherins (E-, VE-, N- and P-cadherin) are the central transmembrane proteins of the adherens junction complex and mediate Ca^2+^-dependent homophilic intercellular interactions. β-catenin and plakoglobin/γ-catenin bind to cadherin intracellular domains in a mutually exclusive manner and α-catenin in turn binds to β-catenin [8]. p120-catenin (p120ctn) on the other hand binds to a different region of cadherins to β-catenin [9],[10],[11],[12],[13],[14]. The composition of proteins of the adherens junction complex depends on the cell type and cadherin expression. α-catenin provides an important link between adherens junctions and the actin cytoskeleton. Its interaction with actin filaments however, appears to be mutually exclusive to its binding to β-catenin suggesting an indirect link between junctions and the actin cytoskeleton [15],[16].

p120ctn has been found to regulate adherens junction stability *in vitro* [17],[18],[19] and *in vivo* [20],[21],[22]. Its depletion by RNAi leads to a decrease in adherens junction proteins and abolishes cell-cell adhesion [17], in part by increasing cadherin endocytosis and degradation [19],[23]. p120ctn also affects the activity of the small GTPases RhoA, Rac1 and Cdc42 in some cell types [24],[25],[26],[27], which play a central role in regulating cytoskeletal dynamics, the formation and maintenance of adherens junctions and cell migration [28],[29]. p120ctn can therefore link adherens junctions and Rho GTPases.

The effects of p120ctn on cell migration and invasion vary depending on the cell type, assay conditions and types of cadherins expressed [30],[31],[32]. It is not clear to what extent the effects of p120ctn are mediated through adherens junctions or Rho GTPases. To address this, we have knocked down p120ctn in DU145 prostate cancer cells and MCF10A mammary epithelial cells, both of which normally have adherens junctions containing E-cadherin and P-cadherin. In both cell types depletion of p120ctn leads to disruption of cell-cell contacts, downregulation of adherens junction proteins and increased cell motility. Interestingly, the knockdown of p120ctn did not affect activity levels of Rho GTPases Rac1 and RhoA but led to an increase in active Rap1. For the first time we have knocked down cadherins as well as catenins and show that P-cadherin, β-catenin and α-catenin but not E-cadherin depletion mimic the effects of p120ctn suppression and lead to a loss of cell-cell contacts and enhanced invasion.

## Results

### Suppression of p120ctn expression leads to downregulation of cell-cell adhesion proteins and disruption of cell-cell contacts

**Figure 1:**
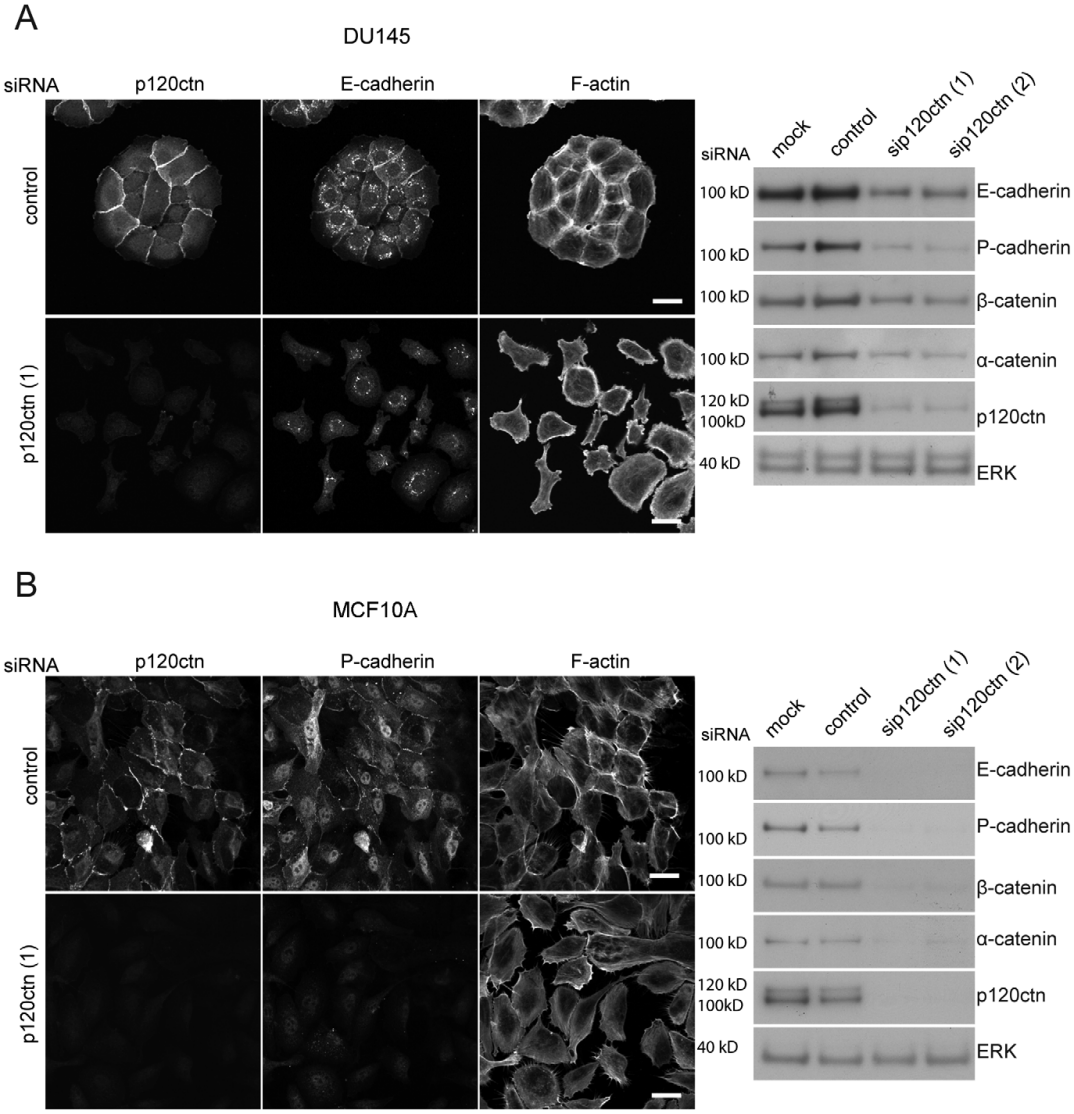
p120ctn depletion disrupts cell-cell contacts in DU145 and MCF10A cells. DU145 (A) and MCF10A (B) cells were transfected with siRNAs for p120ctn (p120ctn (1), (2)), control siRNA or with transfection reagent only (mock). After 72 h, cells were fixed and stained for F-actin, p120ctn and E-cadherin or P-cadherin (left panels), or lysed and analysed by immunoblotting for the indicated cell-cell adhesion molecules and p120ctn knockdown efficiency (right panels). Scale bars  = 25 µm. ERK was used as a loading control for immunoblots.

To investigate the effects of p120ctn on cell-cell adhesion, we compared the effects of p120ctn depletion in two different cell lines that have adherens junctions, the human prostate cancer cell line DU145 and the human mammary epithelial cell line MCF10A. DU145 and MCF10A cells formed cell-cell contacts and expressed both E- and P-cadherin, which localized to cell-cell contacts (Fig. 1A, B). DU145 and MCF10A cells predominantly express two p120ctn isoforms that, based on their molecular weight, are predicted to be isoforms 1 and 3 [33] with isoform 3 (lower band) being expressed at slightly higher levels (Fig. 1).

**Figure 2:**
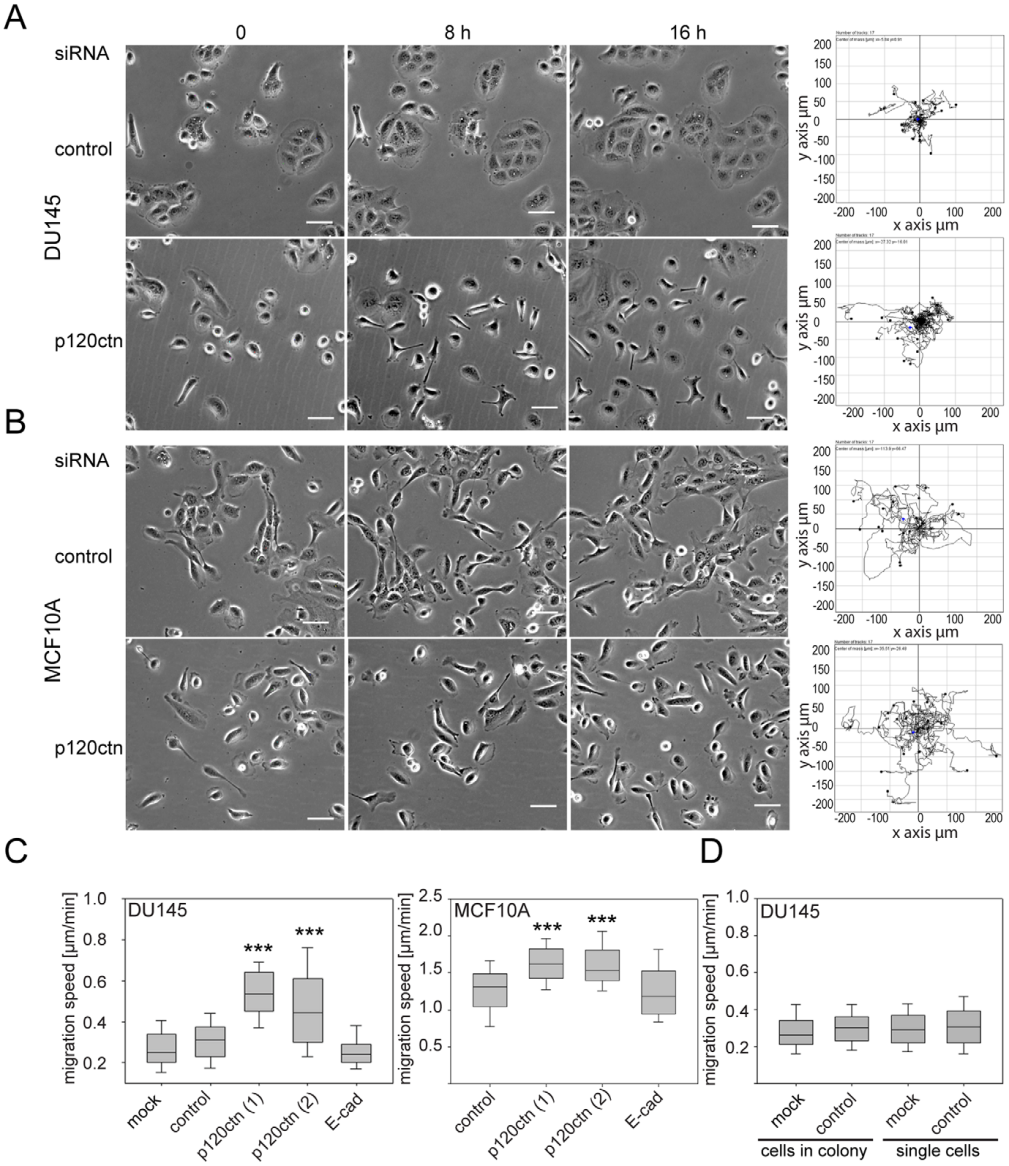
p120ctn depletion increases motility in DU145 and MCF10A cells. DU145 (A, C, D) and MCF10A (B, C) cells were transfected with siRNAs for p120ctn (p120ctn (1), (2)), siRNA for E-cadherin (E-cadherin), control siRNA (control) or with transfection reagent only (mock). After 56 h, cells were monitored by time-lapse microscopy, acquiring an image every 10 min over a period of 16 h. Representative examples of phase-contrast images of cells from the movies (left panels) and migration tracks (right panels; start point of each cell is plotted at intersection of x- and y-axes) are shown for control-transfected and p120ctn-depleted DU145 (A) and MCF10A (B) cells. Scale bars  = 50 µm. (C, D) Migration speeds are depicted as box and whisker plots showing median, interquartile range (boxes) and highest and lowest values (whiskers). (C) Migration speeds of DU145 and MCF10A cells. (D) Migration speeds of DU145 control cells in colonies compared to single cells. Data are from three different experiments, each carried out in duplicate, analysing 15 cells per field (∼90 cells in total). ***p<0.001, compared to control and determined by Student's t-test.

Knockdown of p120ctn with two different siRNAs in DU145 and MCF10A cells induced disruption of cell-cell adhesion resulting in a ’scattered’ phenotype (Fig. 1, Fig. 2). In addition, DU145 cells stably depleted of p120ctn did not have stable adherens junction (Fig. S1). This phenotype was specifically due to p120ctn depletion, since expression of shRNA-resistant mouse p120ctn (GFP-fusion) rescued junction formation, as determined by localization of p120ctn and E-cadherin (Fig. S1).

Both E-cadherin and P-cadherin as well as β- and α-catenin were downregulated in p120ctn-depleted cells. Downregulation of cadherins correlated with the extent of p120ctn depletion: at 72 h after transfection with siRNAs targeting p120ctn, p120ctn levels were lower than at 48 h, and E-cadherin levels were lower at 72 h than 48 h (data not shown).

p120ctn-depleted cells were however not likely to have undergone epithelial to mesenchymal transition (EMT) as levels of EMT marker proteins such as vimentin were not increased compared to control-transfected DU145 cells (data not shown).

### p120ctn regulates migration of DU145 and MCF10A cells

To test whether cell migration was affected by p120ctn depletion, cells were monitored by time-lapse microscopy over 16 h (Fig. 2A, B) and tracked to determine their migration speed. p120ctn-depleted DU145 and MCF10A cells both migrated faster than control cells (Fig. 2, Movies S1, S2, S3 and S4). By contrast, depletion of E-cadherin did not alter cell migration speed (Fig. 2C), even though its expression level was reduced by p120ctn knockdown (Fig. 1). The migration speed of control cells was determined from cells localised within cell colonies (Fig. 2C), but some cells in the population migrated as single cells (Movies S1 and S3). However, there was no difference in the migration speed of DU145 cells in colonies or single cells (Fig. 2D), ruling out the possibility that p120ctn-depleted cells migrated faster because they were predominantly single cells rather than in colonies.

**Figure 3:**
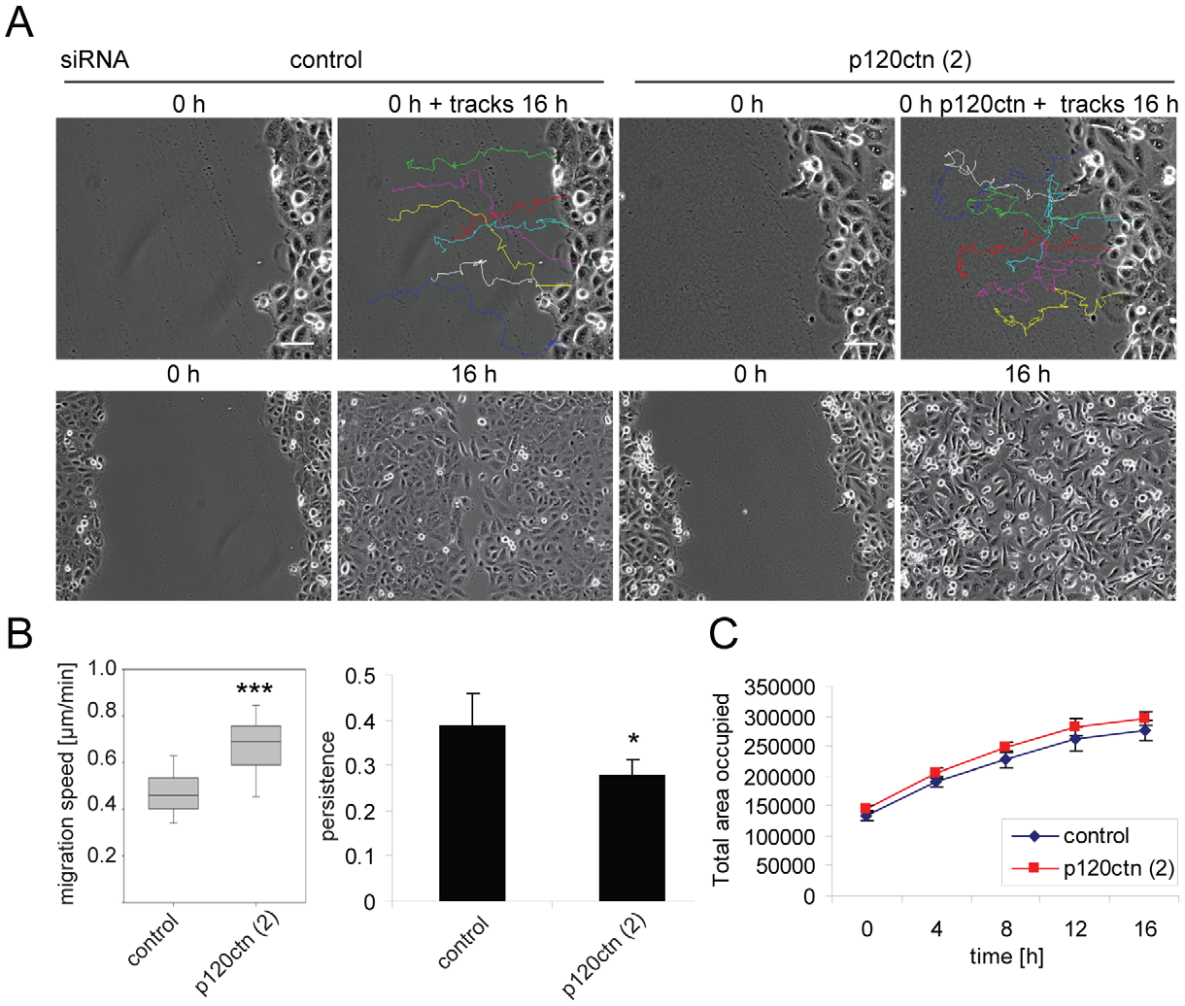
p120ctn depletion decreases directional persistence of cells migrating into a scratch wound. DU145 cells were transfected with siRNAs for p120ctn (p120ctn (2)) and control siRNA (control). After 56 h, a confluent monolayer was wounded using a pipette tip and cells monitored by time-lapse microscopy, acquiring an image every 10 min over a period of 16 h. (A) Phase-contrast images of cells from the first frame of each movie are shown without or with representative tracks of cells (7/image; tracked for 16 h) superimposed (top images). Additionally, scratch wounds of control cells and p120ctn-depleted cells are shown at 0 h and 16 h after wounding (bottom panels). Scale bars  = 50 µm. (B) Migration speed (left) and persistence (right; displacement/track length) were determined. Migration speeds are depicted as box and whisker plots showing median, interquartile range (boxes) and highest and lowest values (whiskers). (C) Area occupied by cells in scratch wounds was determined from phase-contrast images taken at different timepoints from movies. Data are from three different experiments, each carried out in duplicates, analysing 15 cells per field (∼90 cells in total). *p<0.05; ***p<0.001, compared to control and determined by Student's t-test.

Cell migration was also analysed in scratch-wound assays on confluent monolayers of DU145 cells. Most p120ctn-depleted cells migrated as individual cells into the wound, whereas control cells migrated as a sheet, maintaining cell-cell contacts (Fig. 3A, Movies S5, S6). p120ctn-depleted cells migrated faster than control cells (Fig. 3B). However, p120ctn-depleted cells showed a decrease in persistence (Fig. 3B), which indicates that they change direction more frequently. Thus, despite the increased migration speed, wound closure time was similar to control-transfected cells (Fig. 3C).

### Suppression of p120ctn expression leads to increased Rap1 activity

**Figure 4:**
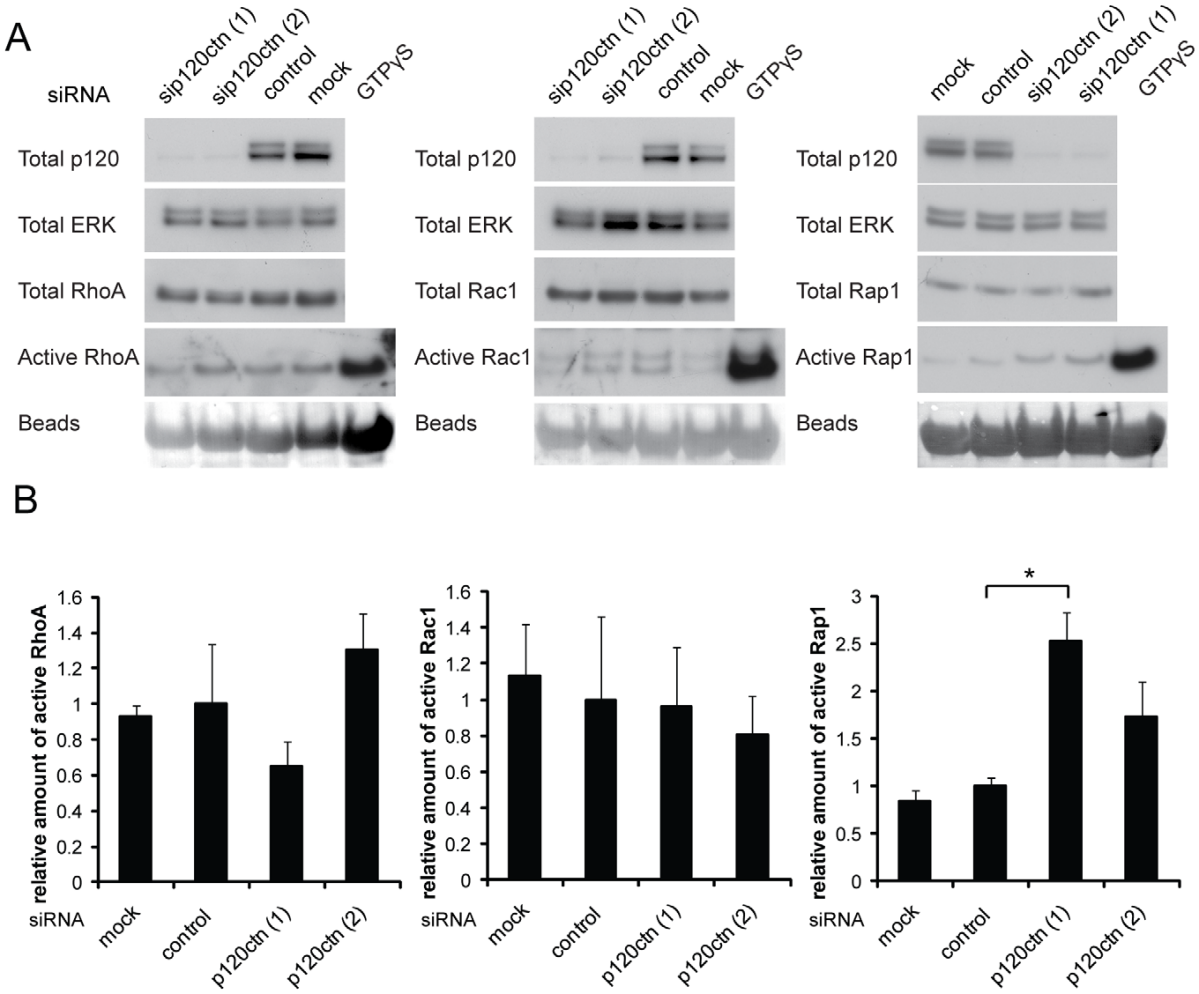
p120ctn depletion does not affect RhoA or Rac1 activity but increases Rap1 activity. DU145 cells were transfected with siRNAs for p120ctn (p120ctn (1), (2)), control siRNA (control) or with transfection reagent only (mock). After 72 h cells were lysed and incubated with GST-Rhotekin-RBD, GST-PAK1-PBD or GST-RalGDS-RBD on glutathione beads to pull down active RhoA, Rac1 and Rap1 respectively. Lysates of mock-transfected cells incubated with GTPγS to preload GTPases were used as a positive control for pulldown assays. (A) Example immunoblots for GTPase activity assays. ERK levels on immunoblots were used as a loading control. Ponceau staining of immunoblots shows the levels of GST-fusion proteins. (B) Graphs show data from 3 (RhoA, Rac1) or 4 (Rap1) independent experiments and results are compared to total RhoA, Rac1, Cdc42 and normalised to control. Error bars represent SEM. * p<0.05, compared to control and determined by Student's t-test.

Regulation of Rho GTPase activities by p120ctn has previously been correlated with increased cell motility [25], [26]. However, no differences in the levels of active RhoA, Rac1 or Cdc42 were observed between p120ctn-depleted and control-transfected DU145 cells (Fig. 4A, B; data for Cdc42 not shown). Since p120ctn knockdown induced a loss of cell-cell contacts and the GTPase Rap1 has previously been associated with stabilisation of E-cadherin at the plasma membrane and to be activated following E-cadherin disengagement [34], levels of active Rap1 were analysed. p120ctn-depleted DU145 cells showed increased levels of Rap1 activity (Fig. 4A, B).

### Depletion of cadherins, ß-catenin or α-catenin does not affect expression levels of other adherens junction proteins

**Figure 5:**
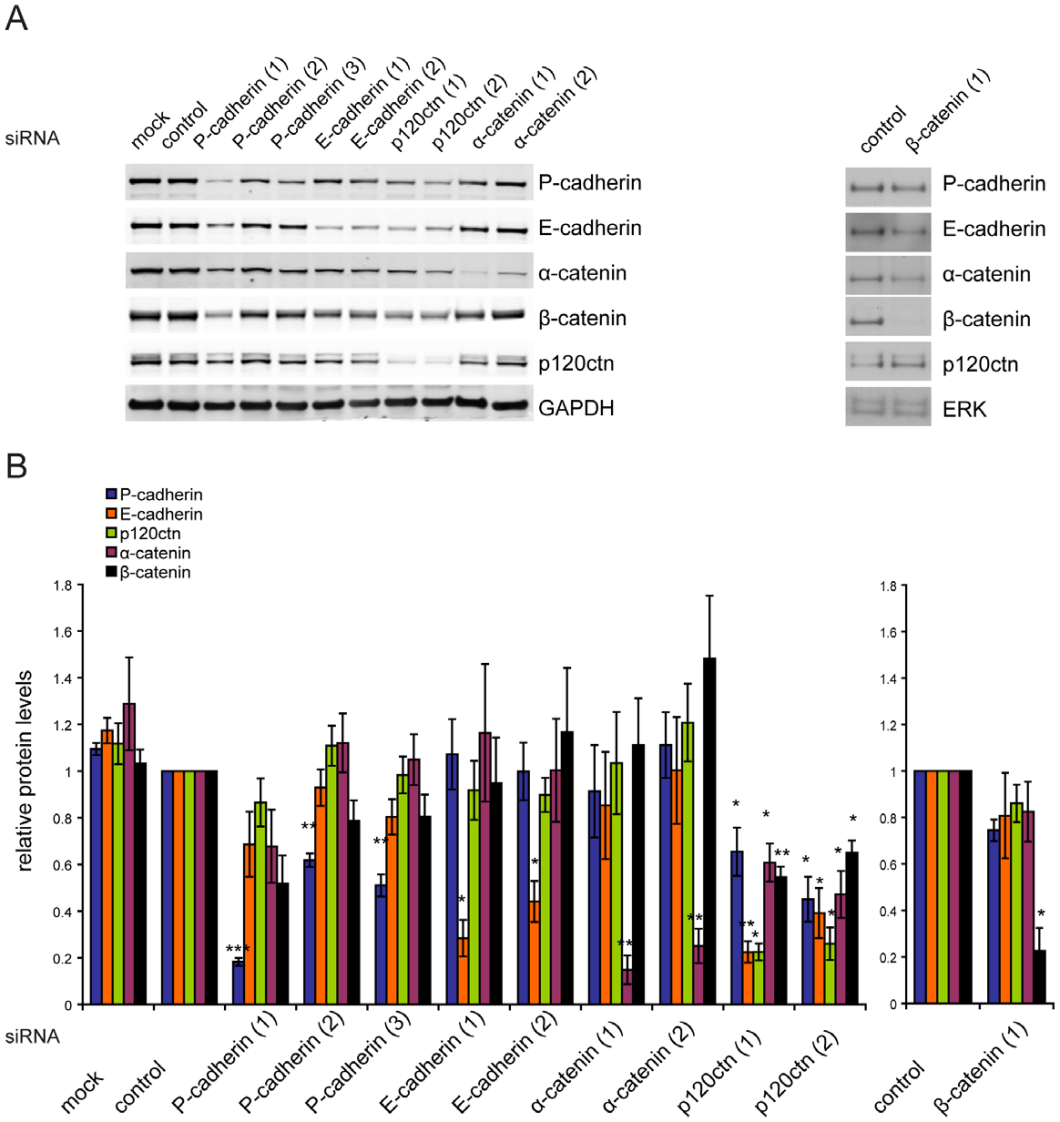
Effects of knocking down adherens junction proteins on expression of other junctional proteins. DU145 cells were transfected with the indicated siRNAs for E-cadherin, P-cadherin, α-catenin and β-catenin, or with control siRNA (control), or transfection reagent only (mock). (A) After 72 h cells were lysed and protein levels of indicated proteins analysed by immunoblotting. Total ERK or GAPDH protein levels were used as a loading control. (B) Graphs show protein levels of P-cadherin, E-cadherin, α-catenin and p120ctn following knockdown of the indicated proteins , quantified by Odyssey scanning of immunoblots (left) or β-catenin knockdown, quantified by densitometry (right), from 4 independent experiments. Results are normalised to control. Bars represent SEM. *** p<0.001, ** p<0.01, * p<0.05, compared to control and determined by Student's t-test.

Since p120ctn depletion reduced the levels of the adherens junction proteins E-cadherin, P-cadherin, ß-catenin and α-catenin (Fig. 1), we investigated the effects of depleting each of these proteins on adherens junctions. DU145 cells were transfected with 2 or 3 different siRNA oligos for E-cadherin, P-cadherin, β-catenin, α-catenin and p120ctn (Fig. 5A).

Expression levels of each of these proteins were then determined by quantification of immunoblots. Levels of P-cadherin, β-catenin, α-catenin and p120ctn proteins were not affected by E-cadherin depletion (Fig. 5A, B). Knockdown of P-cadherin slightly reduced levels of E-cadherin, ß-catenin and α-catenin but not p120ctn, but this was only observed for siRNA oligo (1) that gave the strongest (>80%) depletion of P-cadherin. Two other oligos that reduced P-cadherin levels by ∼50% did not affect the levels of other junctional proteins. ß-catenin or α-catenin depletion did not alter the levels of other adherens junction proteins significantly (Fig. 5). It is thus only p120ctn depletion that results in a decrease in the levels of all the other cell-cell adhesion molecules (Fig. 5B).

### Suppression of p120ctn, P-cadherin, β-catenin and α-catenin but not E-cadherin leads to disruption of cell-cell contacts and enhances cancer cell invasion

**Figure 6:**
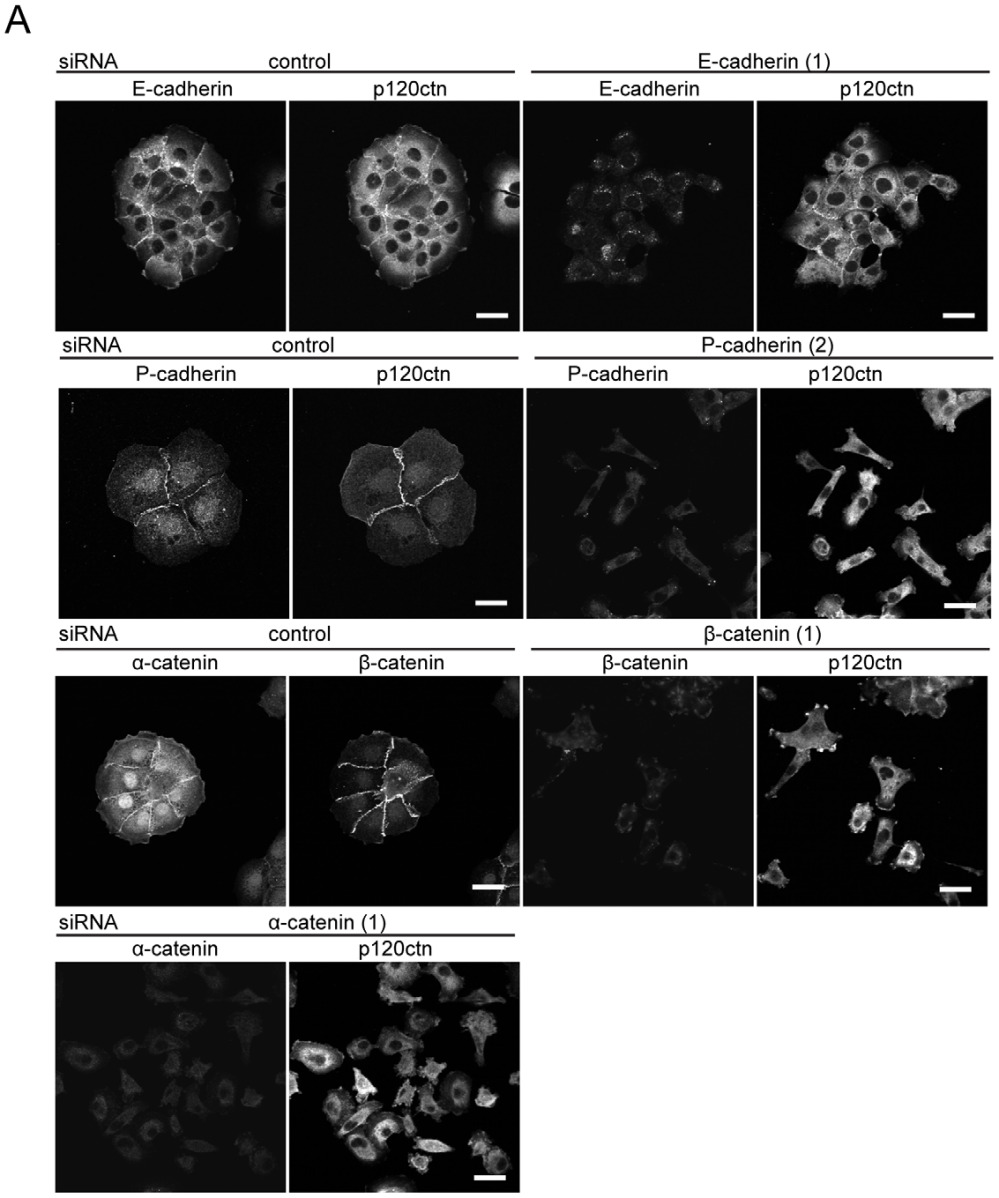
Depletion of P-cadherin, β-catenin and α-catenin but not E-cadherin leads to the disruption of cell-cell contacts. (A) DU145 cells were transfected with the indicated siRNAs for E-cadherin, P-cadherin, β-catenin, α-catenin, control siRNA (control) or with transfection reagent only (mock). After 72 h, cells were fixed and stained for P-cadherin, E-cadherin, β-catenin, α-catenin and p120ctn to correlate knockdown phenotypes with expression of knocked-down proteins. Scale bars  = 25 µm.

Since downregulation of p120ctn led to a loss of cell-cell junctions and an increase in cell migration speed, the effects of depleting other adherens junction proteins on cell-cell adhesion and cell motility were investigated. Knockdown of E-cadherin, P-cadherin, ß-catenin and α-catenin by RNAi was highly efficient both within colonies and in single cells, as observed by immunofluorescence, but did not discernibly affect p120ctn levels (Fig. 6).

**Figure 7:**
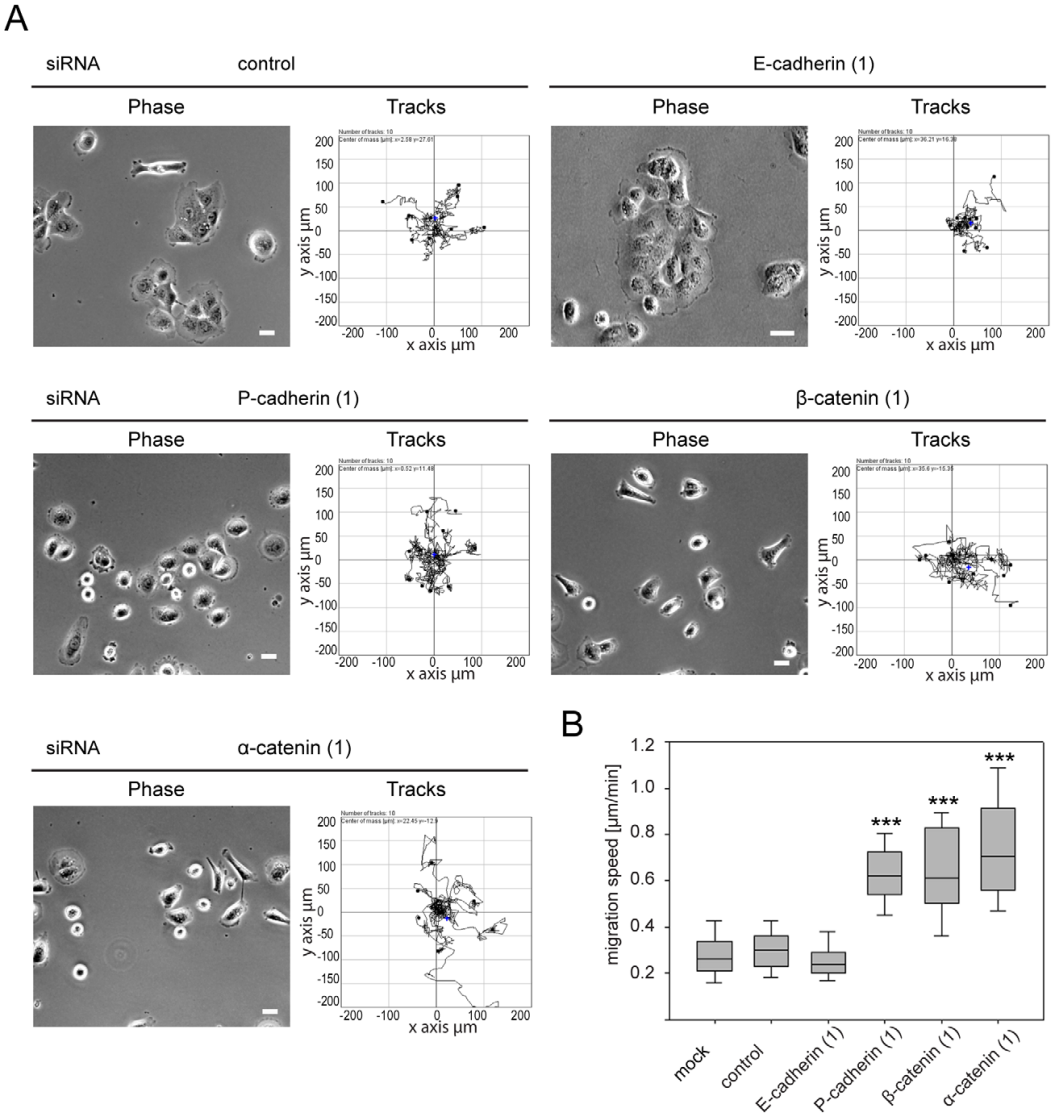
Effects of E-cadherin, P-cadherin, β-catenin and α-catenin depletion on cell migration. DU145 cells were transfected with the indicated siRNAs for E-cadherin, P-cadherin, β-catenin, α-catenin and p120ctn, with control siRNA (control) or transfection reagent only (mock). 56 h after transfection, cells were monitored by time-lapse microscopy, acquiring a phase-contrast image every 10 min over a period of 16 h. Representative examples of phase contrast images at the start and migration tracks after 16 h are shown. Scale bars  = 25 µm. (B) Migration speed was determined using ImageJ Software. Analysis of migration speed is depicted as box and whisker plots showing median, interquartile range (boxes) and highest and lowest values (whiskers). Data are from at least 50 cells from three different experiments. ***p<0.001, compared to control and determined by Student's t-test.

The knockdown of P-cadherin, β-catenin and α-catenin by each of 2 different siRNAs (or 3 for P-cadherin) led to disruption of cell-cell contacts, similar to p120ctn depletion (Fig. 6, Fig. S2, Fig. S3). Notably, all three siRNAs 1, 2 and 3 targeting P-cadherin induced loss of cell-cell contacts, although they reduced P-cadherin levels by different amounts (Fig. 5, Fig. 6, Fig. S3). By contrast, knockdown of E-cadherin in DU145 cells did not induce a loss of cell-cell contacts (Fig. 2C, Fig. 6, Fig. 7).

**Figure 8:**
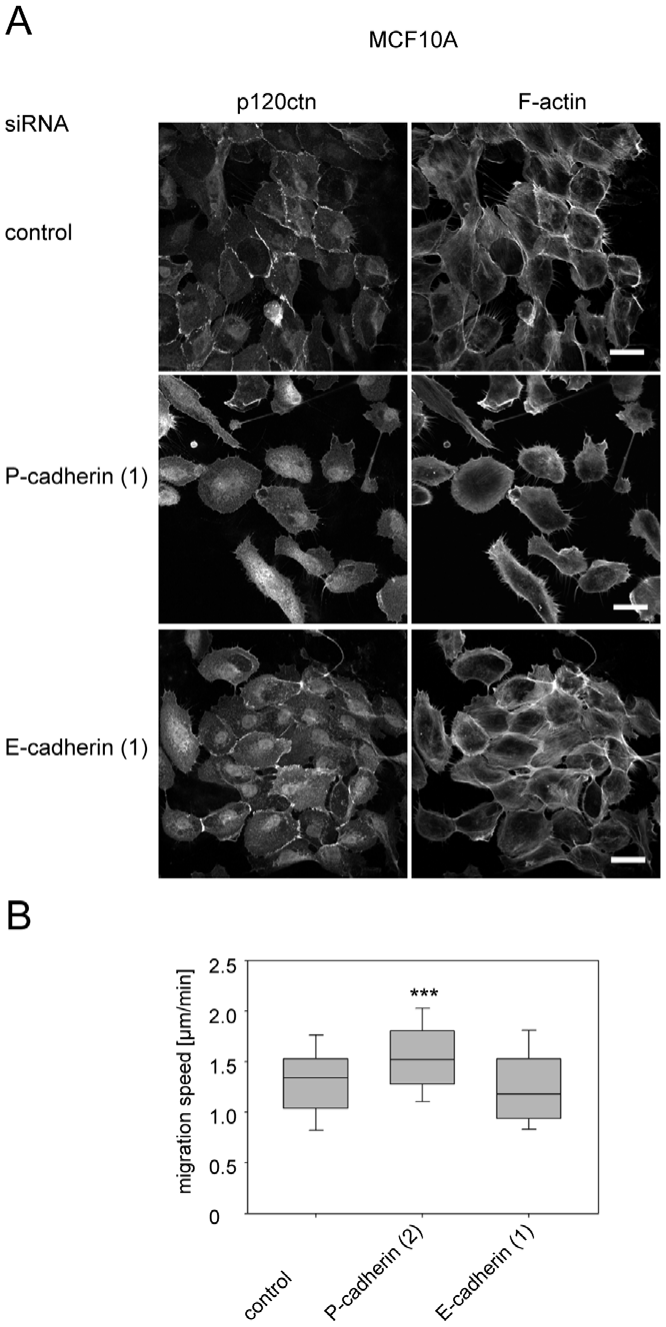
Depletion of P-cadherin but not E-cadherin disrupts cell-cell contacts and increases migration in MCF10A cells. (A) MCF10A cells were transfected with the indicated siRNAs for E-cadherin, P-cadherin or with control siRNA (control). After 72 h, cells were fixed and stained for F-actin and p120ctn. Scale bars  = 25 µm. (B) 56 h after transfection, cells were monitored by time-lapse microscopy, acquiring a phase-contrast image every 10 min over a period of 16 h. Migration speed was determined using ImageJ Software. Analysis of migration speed is depicted as box and whisker plots showing median, interquartile range (boxes) and highest and lowest values (whiskers). Data are from at least 50 cells from three different experiments. ***p<0.001, compared to control and determined by Student's t-test.

Cells depleted of each adherens junction protein were tracked from timelapse movies to determine their migration speed (Fig. 7). Like p120ctn depletion, knockdown of P-cadherin, β-catenin and α-catenin led to an increase in cell migration speed, similar to results obtained with p120ctn depletion, whereas E-cadherin depletion did not alter migration speed (Fig. 7). Similarly P-cadherin but not E-cadherin depletion in MCF10A cells led to disruption of cell-cell contacts and increased migration speed (Fig. 8).

**Figure 9:**
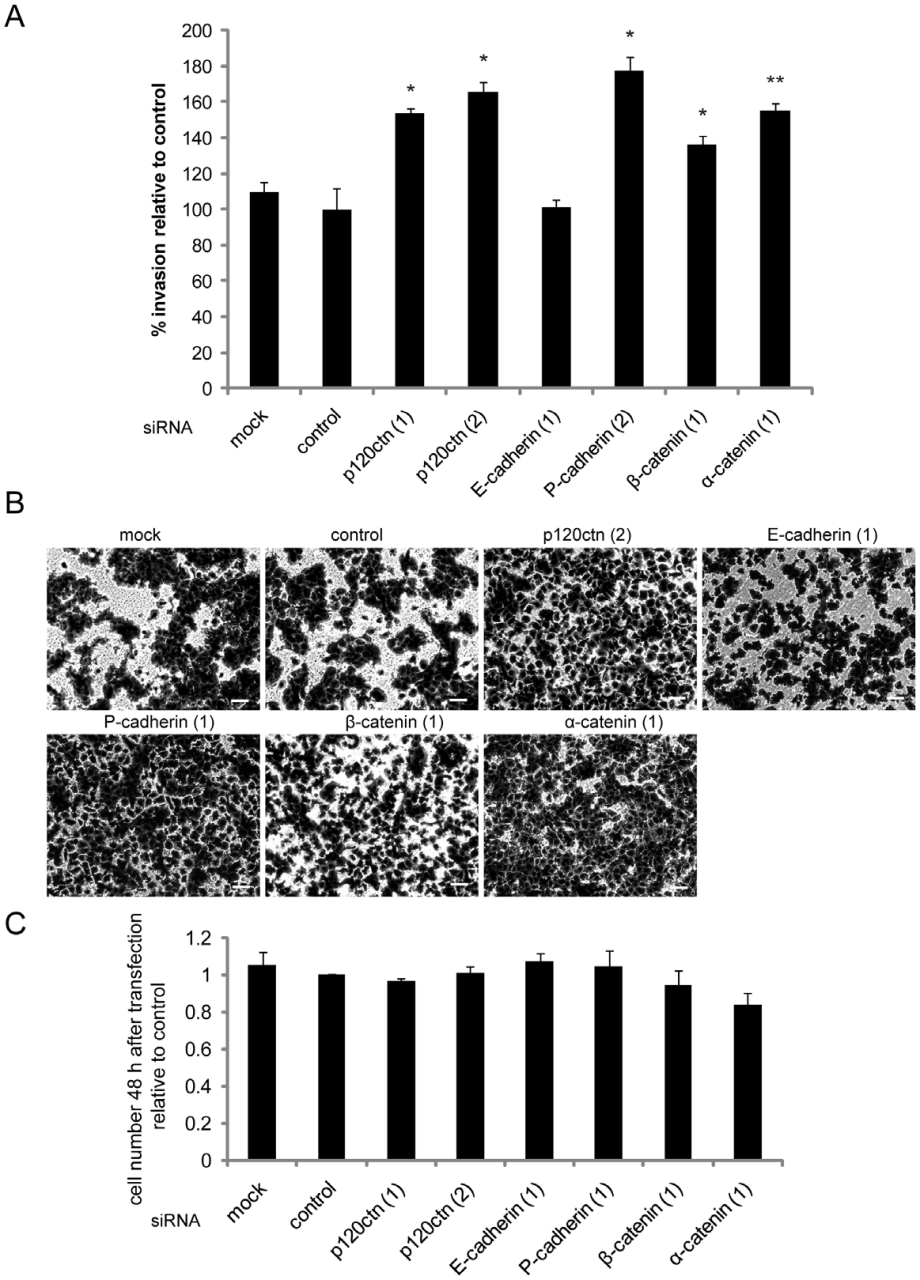
Depletion of p120ctn, P-cadherin, β-catenin and α-catenin but not E-cadherin increases invasion. DU145 cells were transfected with the indicated siRNAs for p120ctn, P-cadherin, E-cadherin, α-catenin and β-catenin, with control siRNA (control) or transfection reagent only (mock). After 55 h cells were seeded onto Transwell membrane inserts containing a layer of Matrigel. FCS was used as a chemoattractant in the bottom chamber. Assays were stopped after 17 h by fixing cells on the bottom of the membrane inserts using crystal violet. Images of eight different fields of view were acquired and cells in all images counted and analysed. (A) Graphs show data from three independent experiments, each carried out in triplicate. Results are normalised to control. Bars represent SEM. **p<0.01, *p<0.05, compared to control, determined by Student's t-test. (B) Representative images of cells on the bottom of the Transwell membranes. Scale bars  = 50 µm. (C) 48 h after transfection cells were counted and compared to sicontrol. Graphs show data from at least three independent experiments. Results are normalised to control. Bars represent SEM.

Based on the changes in cell-cell adhesion and migration speed observed, the effects of p120ctn, E-cadherin, P-cadherin, β- and α-catenin on invasion through Matrigel was investigated. Knockdown of p120ctn with two different siRNAs increased invasion through Matrigel whereas E-cadherin depletion did not affect invasion (Fig. 9A, B). The knockdown of P-cadherin on the other hand led to an increase in invasion similar to p120ctn depletion (Fig. 9). The depletion of β-catenin as well as α-catenin induced an increase in invasion similar to p120ctn and P-cadherin. To rule out the possibility that differences in the number of cells on the bottom of Matrigel-coated Transwells were due to altered cell proliferation, cells were counted 48 h after they had been transfected with siRNAs. No changes in cell numbers compared to control siRNA-transfected cells were observed (Fig. 9C). In conclusion, knockdown of P-cadherin, β-catenin and α-catenin but not E-cadherin induced a disruption of cell-cell contacts and an increase in cancer cell migration and invasion similar to the knockdown of p120ctn.

## Discussion

p120ctn expression is frequently lost or downregulated in a large variety of human cancers [35] and decreased p120ctn levels correlate with the tumour grade [36],[37],[38],[39]. We have shown here that the depletion of p120ctn in E-cadherin and P-cadherin-expressing DU145 and MCF10A cells disrupts cell-cell contacts, and increases migration and invasion of DU145 cells, supporting a model where p120ctn acts as an invasion or metastasis suppressor [48]. These effects correlated with decreased expression of the adherens junction proteins E-cadherin, P-cadherin, ß-catenin and α-catenin. However, only downregulation of P-cadherin and not E-cadherin was able to induce a similar phenotype to p120ctn depletion, indicating that E-cadherin is not required to maintain cell-cell adhesion in DU145 or MCF10A cells.

In contrast to our results, p120ctn has been reported to be required for invasion of E-cadherin-deficient cells in part by stabilising the mesenchymal cadherins N-cadherin or cadherin-11, which are known to promote invasion [32]. On the other hand the collective invasion of E- and P-cadherin-expressing A431 cells was inhibited when cell-cell contacts were disrupted either by p120ctn knockdown or the simultaneous knockdown of E-cadherin and P-cadherin [35]. In these studies HGF or EGF, respectively, were used as chemoattractants in invasion and migration assays, whereas we used serum. One possibility is that p120ctn could specifically contribute to HGF/EGF-induced migration or invasion as it has previously been linked to HGF- or EGF-induced cell scattering [40].

p120ctn has been shown to stimulate Rac and/or Cdc42 activity and/or inhibit RhoA activity in cells lacking E-cadherin, including fibroblasts and cancer cell lines [32],[41]. In contrast, we found that in DU145 cells that express P- and E-cadherin but not N-cadherin, the depletion of p120ctn did not affect activity of RhoA and Rac1. Similarly, in E-cadherin/P-cadherin-expressing A431 cells, p120ctn depletion did not alter RhoA/Rac1 activity [31].

Taken together, these results indicate that the effects of p120ctn on Rho GTPase activities are cell type-specific. This could be due to differences in the composition of p120ctn complexes, for example complexes containing GEFs that could activate Rac1/Cdc42 or GAPs to downregulate RhoA [26],[41]. Interestingly, in MDA-MB-231 breast cancer cells, activation of Rac by p120ctn requires cadherin binding [32], suggesting that mesenchymal cadherins could specifically contribute to Rac activity. Although Rho GTPase activity was not affected by p120ctn, we found that Rap1 activity is increased in p120ctn-depleted DU145 cells. Rap1 is an important regulator of cell-cell junctions, and is activated by E-cadherin engagement [42], [43]. Disruption of adherens junctions by extracellular calcium depletion also induces an increase in Rap1 activity [44]. Our results with p120ctn depletion are consistent with the hypothesis that endocytosis of E-cadherin increases Rap1 activity [44].

Surprisingly, the knockdown of P-cadherin, β- and α-catenin but not E-cadherin led to the disruption of cell-cell contacts. Presumably P-cadherin substitutes for E-cadherin to maintain cell-cell contacts, but not E-cadherin for P-cadherin. This hypothesis is supported by studies in MDCK cells, where it has been shown that E-cadherin is important only for the establishment but not maintenance of cell-cell contacts, whereas α-catenin was required to maintain cell-cell contacts [45]. In addition, P-cadherin depletion alone was reported to induce loss of cell-cell contacts and downregulation of E-cadherin levels in MCF10A cells [46] and overexpression of P-cadherin in MDA-MB-231 breast cancer cells reduced cell migration and invasion [47]. In cancer most studies have concentrated on the link between E-cadherin downregulation and increased cancer aggressiveness and invasion, although there are also indications that P-cadherin affects tumour progression. For example, P-cadherin levels are downregulated during melanoma progression [48] and P-cadherin is often lost in prostate cancers [49]. In DU145 cells, we found that P-cadherin depletion induces loss of cell-cell junctions without significantly affecting the levels of other adherens junction proteins, indicating that it is the major cadherin required for cell-cell adhesion in these cells.

The knockdown of β- and α-catenin also induced disruption of cell-cell contacts and an increase in invasion but did not change P-cadherin or p120ctn protein levels. It is possible that they affect the localization of P-cadherin and/or p120ctn rather than levels. β- and α-catenins have been reported to affect cadherin-mediated adhesion [15],[16], and the depletion of β-catenin in an E-cadherin-deficient cell line led to a decrease in invasion [50], but so far little is known about their roles in migration and invasion. β-catenin also has an important role in Wnt-signalling and cancer cell proliferation which is thought to be independent of its cadherin function [51].

Our results demonstrate that p120ctn regulates the expression levels of ß- and α-catenin as well as cadherins in DU145 cells. Since we have shown that P-cadherin levels are more important than E-cadherin in maintaining cell-cell adhesion in DU145 and MCF10A cells, we postulate that the increased migration and invasion we observe following p120ctn, ß-catenin or α-catenin knockdown is due either to decreased P-cadherin stability or changes in its localization.

## Materials and Methods

### siRNAs

All siRNAs used were obtained from Dharmacon (Fisher Scientific UK, Loughborough, UK). The following ON-TARGET *plus* single oligos were used targeting p120ctn (CTNND1 (1) 5′-UAGCUGACCUCCUGACUAA-3′; (2) 5′-GGACCUUACUGAAGUUA-3′), E-cadherin (CDH1 (1) 5′-GGCCUGAAGUGACUCGUAAU-3′; (2) 5′GAGAACGCAUUGCCACAUA-3′), P-cadherin (CDH3 (1) 5′GUGACAACGUCUUCUACUA-3′; (2) 5′-GAGGGUGUCUUCGCUGUAG-3′; (3) 5′-GAAAUCGGCAACUUUAUAA-3′), β-catenin (CTNNB1 (1) 5′-GCGUUUGGCUGAACCAUCA-3′) and α-catenin (CTNNA1 (1) 5′-GAUGGUAUCUUGAAGUUGA-3′; (2) 5′-GUGGAUAAGCUGAACAUUA-3′) . Non-targeting siRNA was used as a control in all experiments (ON-TARGET control #1; 5′-UGGUUUACAUGUCGACUAA-3′).

### Cell culture and transfections

DU145 prostate cancer cells were grown in RPMI supplemented with 10% FCS, 100 IU/ml penicillin and 100 µg/ml streptomycin. MCF10A mammary epithelial cells were grown in DMEM/F12 supplemented with 5% horse serum, 100 IU/ml penicillin, 100 µg/ml streptomycin, 20 ng/ml EGF, 0.5 µg/ml hydrocortisone, 100 ng/ml cholera toxin and 5 µg/ml insulin. Cells were seeded at 1.8×10^5^ per well in 24-well plates and reverse transfected with siRNAs (final concentration 20 nM) in antibiotic-free medium using Lipofectamine 2000 (Invitrogen, Paisley, UK), according to the manufacturer's instructions. After 6–8 h, cells were reseeded at 2×10^4^ cells per well (DU145) or 10^4^ cells per well (MCF10A) for immunofluorescence and time-lapse microscopy and at 1×10^5^ per well (24-well plate) for immunoblotting and scratch-wound assays. For creation of stable p120ctn-depleted DU145 cells, the pSuperior vector system (Oligoengine, Seattle, WA) was used. The sequence of the shRNA oligo targeting human p120ctn was designed as described [52]. A stable cell pool containing the shRNA was selected in medium containing 5 µg/ml puromycin. For rescue experiments murine p120ctn-GFP [26], which is not sensitive to the shRNA targeting human p120ctn, was transiently transfected into DU145 cells using Amaxa nucleofection (Lonza, Cologne, Germany) according to the manufacturer's protocol (www.lonzabio.com).

### Immunoblotting

Cells grown to approximately 80% confluency were washed once with cold PBS and lysed in either cold 2× SDS sample buffer (Invitrogen) directly and homogenized with a 21G needle or in lysis buffer (50 mM Tris pH 7.5, 1% Triton-X-100, 0.5% sodium deoxycholate, 0.1% SDS, 500 mM NaCl, 10 mM MgCl_2_, 2 mM EDTA, 10% glycerol, 1 mM Na_3_VO_4_, 25 mM NaF, complete mini EDTA-free protease inhibitor (Roche, Welwyn Garden City, UK), PhosSTOP phosphatase inhibitor (Roche)) and then cleared by centrifugation. Lysates were separated by SDS-PAGE and subjected to immunoblot analysis. For reprobing, PVDF membranes were incubated with stripping buffer (50 mM Tris HCl (pH 7.5), 100 mM β-mercaptoethanol, 2% SDS) for 20 min at 65°C. Primary antibodies were used at a dilution of 1∶1000: p120ctn (# 610134), E-cadherin (# 610182) were purchased from BD Biosciences (Oxford, UK), P-cadherin (# 05-916), Rac1 (# 05-389) from Upstate (Millipore, Watford, UK), β-catenin (# C2206), α-catenin (# C2081) from Sigma-Aldrich (Dorset, UK), ERK (# sc-94), RhoA (# sc-418) from Santa Cruz Biotechnology (Insight Biotechnology, Wembley, UK) and Rap1A/Rap1B (# 4938) from Cell Signaling (New England Biolabs, Hitchin, UK). Secondary HRP-conjugated mouse (GE Healthcare, Chalfont St Giles, UK) or rabbit antibodies (Dako, Ely, UK) were used at a dilution of 1∶5000. Where indicated, bands on immunoblots were quantified by densitometry using VisionWorksLS analysis software (UVP, Cambridge, UK), automatically subtracting the background from the reading. Alternatively, secondary antibodies conjugated to IRDye 800CW (LI-COR, Cambridge, UK) or Alexa Fluor 680 (Molecular Probes, Invitrogen) were used and membranes scanned and bands quantified with an Odyssey IR scanner (LI-COR) using Odyssey imaging software 2.1.

### RhoA, Rac1 and Rap1 activity assays

GST-Rhotekin-RBD, GST-PAK-PBD and GST-RalGDS-RBD were expressed in *Escherichia coli* and purified as previously described on glutathione beads [53]. Cells were transfected with siRNAs on 10-cm dishes. After 72 h, cells were lysed in 50 mM Tris pH 7.5, 1% Triton-X-100, 0.5% sodium deoxycholate, 0.1% SDS, 500 mM NaCl, 10 mM MgCl_2_, 2 mM EDTA, 10% glycerol, 1 mM Na_3_VO_4_, 25 mM NaF, complete mini EDTA-free protease inhibitor (Roche, Welwyn Garden City, UK), PhosSTOP phosphatase inhibitor (Roche). Lysates were cleared by centrifugation (17,000 g, 30 min, 4°C). A 50 µl aliquot was retained for determination of total levels of each GTPase, and GTPases in 500 µl of control lysate were loaded with GTPγS by incubation with 10 mM EDTA and 100 µM GTPγS for 15 min, followed by 60 mM MgCl_2_ to stop the reaction. This and remaining lysates were incubated with 20 µl of GST-fusion proteins on beads (1 h, 4°C, rotating). Proteins were eluted by boiling in 2× SDS sample buffer, resolved by SDS-PAGE analysed by immunoblotting as described above.

### Immunofluorescence

Cells were seeded on glass coverslips, fixed with 4% paraformaldehyde, permeabilized with 0.1% Triton X-100 and blocked with 2% BSA before staining. Primary antibodies (as listed above; Immunoblotting) were used at a dilution of 1∶200. Alexa Fluor 488, or 546 or 647-conjugated goat anti-mouse IgG or goat anti-rabbit IgG (Invitrogen) were used at a dilution of 1∶1000. F-actin was detected by staining with phalloidin conjugated to Alexa Fluor 488, 546 or 647 (Invitrogen) at a dilution of 1∶1000. Cells were visualised using a Zeiss LSM510 confocal laser-scanning microscope with an EC Plan-Neofluar 40×/1.3 oil DIC M27 objective and ZEN software (Zeiss, Welwyn Garden City, UK). Photoshop and Illustrator (both CS4; Adobe) were used to generate figures.

### Time-lapse microscopy

Time-lapse movies were acquired over a period of up to 16 h using a Nikon TE2000-E microscope with a Plan Fluor 10× or 20× objective (Nikon, Kingston, UK) and a Hamamatsu Orca-ER digital camera. Image series were captured at 37°C and 5% CO_2_ at 1 frame/10 min using Metamorph software (Molecular Devices, Wokingham, UK). Cells were tracked and migration speed (in µm/min) and persistence (displacement/track length, where displacement is the distance from the start to end point for each cell) were determined using ImageJ analysis software (http://rsb.info.nih.gov/ij) and ibidi chemotaxis and migration tool (www.ibidi.com). For scratch-wound assays, area occupied by cells in scratch wounds was determined from phase-contrast images taken at 0 h, 4 h, 12 h and 16 h after wounding using Photoshop (CS4, Adobe).

### Matrigel invasion assays

Matrigel-coated transwell filters with a PET membrane containing 8 µm pores (BD Biosciences) were rehydrated for 2 h at 37°C in medium without supplements. Transfected cells were washed once in PBS, and 10^5^ cells were seeded into the upper chamber of the transwells in antibiotic-free medium containing 0.1% FCS and 0.1% BSA. Medium containing 2% FCS and 0.1% BSA was placed in the bottom chamber as a chemoattractant. Cells were allowed to invade for 17 h. Remaining cells and matrix were then removed from the upper side of the membrane facing the upper chamber. The cells on the bottom part of the membrane were fixed in methanol containing 0.1% crystal violet. Eight separate bright-field images were taken of each transwell filter using a Nikon TE2000-E microscope with a Plan Fluor 10× objective. The cells per image were counted and analysed compared to control transfected cells.

### Statistics

Statistical analysis was carried out where indicated using data from three or more independent experiments. Statistical significance was calculated in Excel (Microsoft) using an unpaired Student's t-test.

## Supporting Information

Figure S1 p120ctn expression rescues the disruption of adherens junctions by p120ctn depletion. Stable p120ctn-knockdown DU145 cells were transiently transfected with plasmids encoding GFP or GFP-p120ctn (wild-type murine cDNA). After 24 h, cells were fixed and stained for E-cadherin. The boxed region (left panel) is shown enlarged in the magnified panels. Scale bars  = 50 µm.
(1.93 MB TIF)

Figure S2 Effects of adherens junction protein depletion using additional siRNA oligos. DU145 cells were transfected with the indicated siRNAs for E-cadherin, P-cadherin, α-catenin, β-catenin or p120ctn. After 72 h, cells were fixed and stained for p120ctn and F-actin.
(2.73 MB TIF)

Figure S3 Disruption of cell-cell adhesion following depletion of P-cadherin using 3 different siRNA oligos. DU145 cells were transfected with the three different siRNAs targeting P-cadherin used in Figure 5. After 72 h, cells were fixed and stained for P-cadherin and F-actin.
(0.93 MB TIF)

Movie S1 DU145 cells, si-control.
(8.62 MB AVI)

Movie S2 DU145 cells, si-p120ctn.
(8.83 MB AVI)

Movie S3 MCF10A cells, si-control.
(8.84 MB AVI)

Movie S4 MCF10A cells, si-p120ctn.
(8.84 MB AVI)

Movie S5 DU145 cells, si-control, scratch wound.
(9.48 MB AVI)

Movie S6 DU145 cells, si-p120ctn, scratch wound.
(9.48 MB AVI)
