# Supplementary figures and images for: p120ctn and P-Cadherin but Not E-Cadherin Regulate Cell Motility and Invasion of DU145 Prostate Cancer Cells
Source: PLoS One. 2010 Jul 27;5(7):e11801. doi: 10.1371/journal.pone.0011801 (PMC2910720; doi:10.1371/journal.pone.0011801)

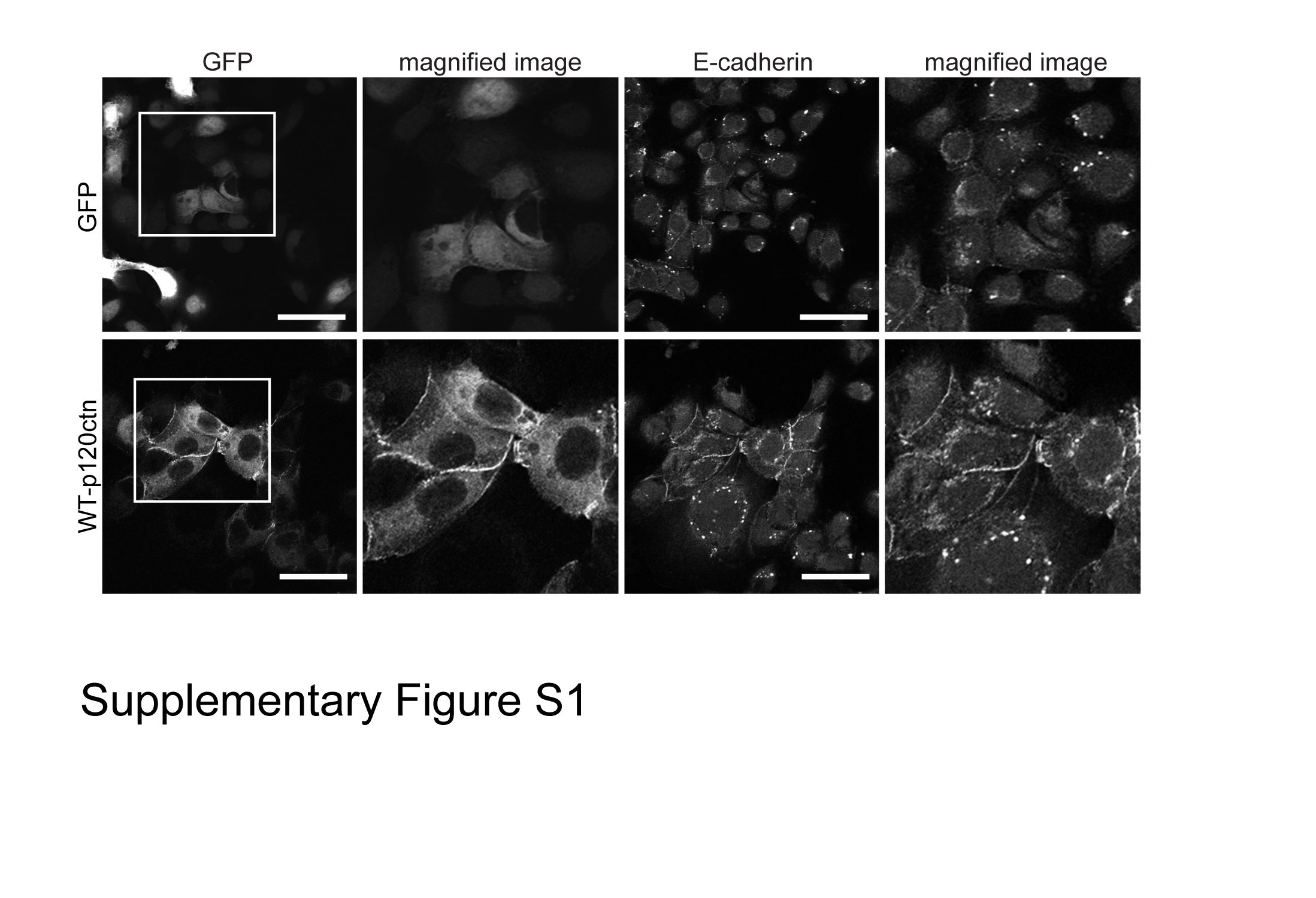

Supplement: Figure S1 — p120ctn expression rescues the disruption of adherens junctions by p120ctn depletion. Stable p120ctn-knockdown DU145 cells were transiently transfected with plasmids encoding GFP or GFP-p120ctn (wild-type murine cDNA). After 24 h, cells were fixed and stained for E-cadherin. The boxed region (left panel) is shown enlarged in the magnified panels. Scale bars = 50 µm. (1.93 MB TIF) [file pone.0011801.s001.tif]

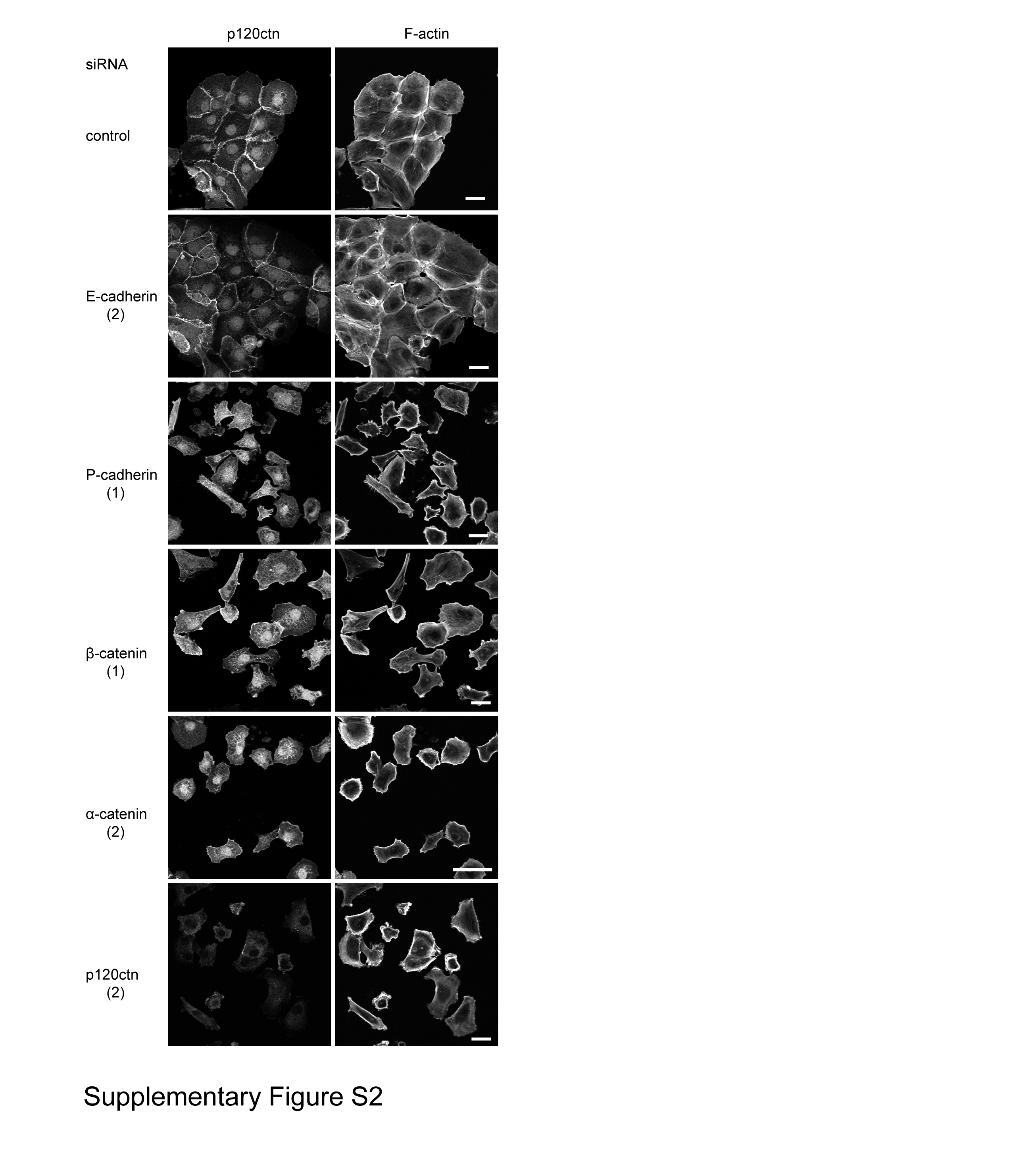

Supplement: Figure S2 — Effects of adherens junction protein depletion using additional siRNA oligos. DU145 cells were transfected with the indicated siRNAs for E-cadherin, P-cadherin, α-catenin, β-catenin or p120ctn. After 72 h, cells were fixed and stained for p120ctn and F-actin. (2.73 MB TIF) [file pone.0011801.s002.tif]

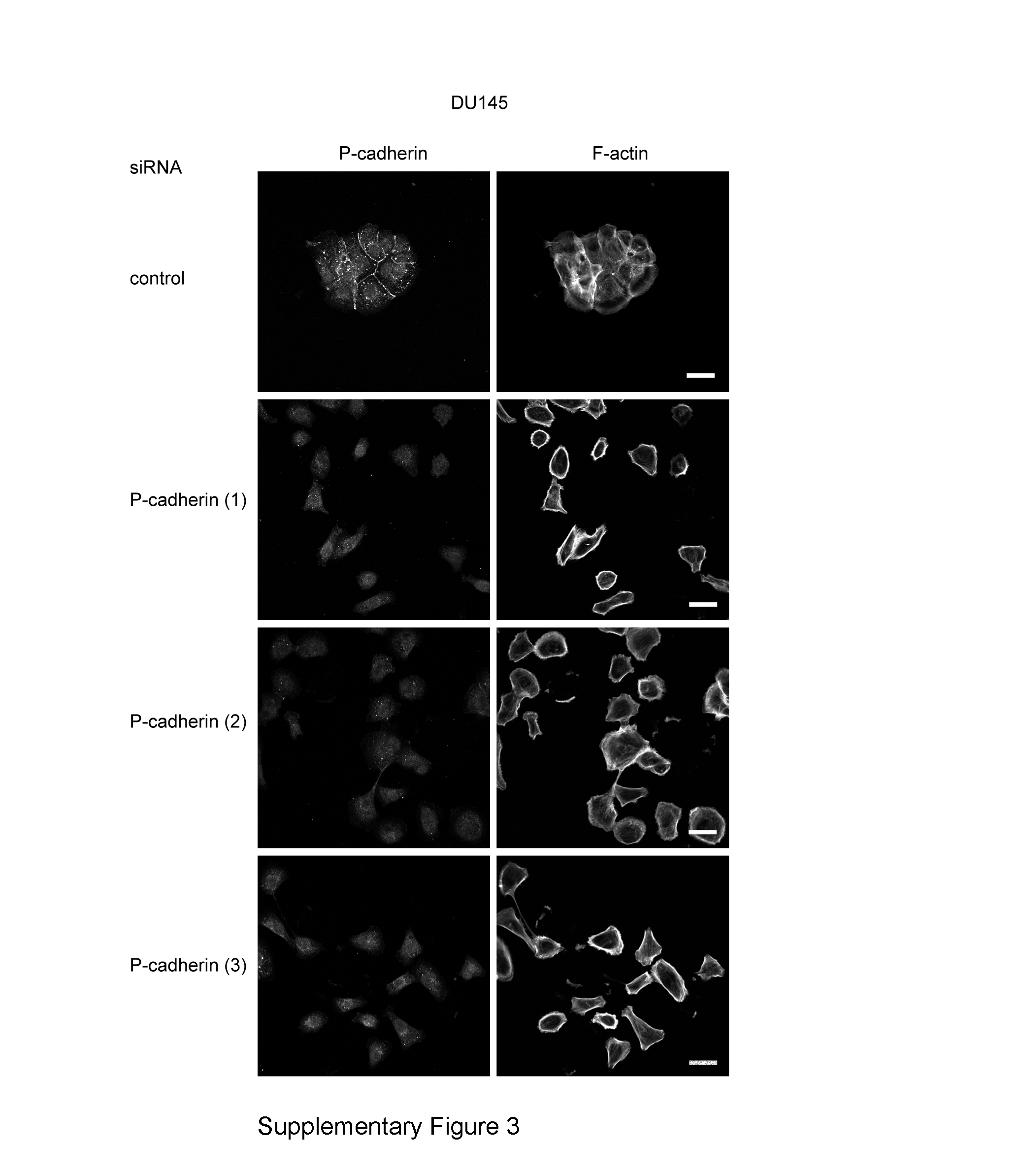

Supplement: Figure S3 — Disruption of cell-cell adhesion following depletion of P-cadherin using 3 different siRNA oligos. DU145 cells were transfected with the three different siRNAs targeting P-cadherin used in Figure 5. After 72 h, cells were fixed and stained for P-cadherin and F-actin. (0.93 MB TIF) [file pone.0011801.s003.tif]
